# Tactile temporal offset cues reduce visual representational momentum

**DOI:** 10.3758/s13414-021-02285-2

**Published:** 2021-03-29

**Authors:** Simon Merz, Christian Frings, Charles Spence

**Affiliations:** 1 Department of Psychology, Cognitive Psychology, University of Trier, Trier, Germany; 2 Department of Experimental Psychology, University of Oxford, Oxford, UK

**Keywords:** Representational momentum, Vision, Touch, Cross-modal, Motion perception

## Abstract

The perception of dynamic objects is sometimes biased. For example, localizing a moving object after it has disappeared results in a perceptual shift in the direction of motion, a bias known as *representational momentum*. We investigated whether the temporal characteristics of an irrelevant, spatially uninformative vibrotactile stimulus bias the perceived location of a visual target. In two visuotactile experiments, participants judged the final location of a dynamic, visual target. Simultaneously, a continuous (starting with the onset of the visual target, Experiments 1 and 2) or brief (33-ms stimulation, Experiment 2) vibrotactile stimulus (at the palm of participant’s hands) was presented, and the offset disparity between the visual target and tactile stimulation was systematically varied. The results indicate a cross-modal influence of tactile stimulation on the perceived final location of the visual target. Closer inspection of the nature of this cross-modal influence, observed here for the first time, reveals that the vibrotactile stimulus was likely just taken as a temporal cue regarding the offset of the visual target, but no strong interaction and combined processing of the two stimuli occurred. The present results are related to similar cross-modal temporal illusions and current accounts of multisensory perception, integration, and cross-modal facilitation.

## Introduction

Localizing dynamic objects is one of the most important tasks performed by our perceptual systems. Knowing and understanding where an object is at any given moment is important for the successful and safe interaction with our environment, and the objects therein. Interestingly, when directly asked to localize a moving object that has just disappeared, participants typically show a systematic bias to overestimate the final location in the direction of motion. This bias, typically referred to as *representational momentum*, has often been evidenced and replicated (Freyd & Finke, 1984; for reviews, see Hubbard, 2005, 2018). While the early research tended to focus mainly on the localization of visual stimuli, the phenomenon has now been documented in the auditory (e.g., Getzmann & Lewald, 2007; Hubbard, 1995; Schmiedchen et al., 2012, 2013) and tactile (Macauda et al., 2018; Merz et al., 2019a, b) modalities as well.

As the *representational momentum phenomenon* has been demonstrated in different sensory modalities, the question arises as to whether the localization of dynamic stimuli is independent of whatever information may happen to be presented in any of the other sensory modalities at around the same time (Hubbard & Courtney, 2010; Merz et al., 2020; Teramoto et al., 2010). As the representational momentum effect depends on the direction of the target, a number of cross-modal studies have investigated if and how the direction of another stimulus, presented in a different sensory modality, changes the localization of the target (Hubbard & Courtney, 2010; Merz et al., 2020). In fact, the evidence suggests that the localization of the final position of a dynamic visual stimulus is not affected by the presentation of a congruent (same direction) or incongruent (different direction) stimulus in either the auditory (Hubbard & Courtney, 2010) or tactile (Merz et al., 2020) modalities. Interestingly, this is not the case when it is the final location of the auditory/tactile stimulus that has to be judged. In the latter case, the localization significantly shifted in the direction of the visual stimulus. This is in line with the typical finding that visual direction perception dominates tactile/auditory perception when it comes to directional bias (cross-modal dynamic capture task; e.g., Soto-Faraco et al., 2003; Soto-Faraco, Spence, & Kingstone, 2004a). Furthermore, this fits with modern accounts of multisensory integration that propose that the information from the different senses is very often combined optimally by weighting the input based on sensory uncertainty/relative resolution (Ernst & Banks, 2002; Ernst & Bülthoff, 2004; though see also Meijer et al., 2019; Rahnev & Denison, 2018).

Interestingly, a number of studies of cross-modal representational momentum have manipulated temporal synchrony rather than directional congruency (Chien et al., 2013; Teramoto et al., 2010). When it comes to temporal perception, auditory and tactile information typically increase the temporal sensitivity of visual information (e.g., as shown by the temporal ventriloquism effect; Morein-Zamir et al., 2003; Vroomen & De Gelder, 2004) or even dominate visual perception (as most prominently shown by the so-called double flash illusion; e.g., Shams et al., 2000; Violentyev et al., 2005). In line with these findings, Teramoto and colleagues documented a cross-modal influence of auditory temporal information on the localization of a dynamic visual target. That is, a spatially uninformative auditory tone was presented with the onset of a dynamic visual target. Whereas the onset of both the auditory and the visual information was synchronous, the offset of the auditory stimulus could occur earlier, later, or else synchronous with the offset of the visual target. Interestingly, when the auditory tone ended shortly before the visual target, the visual forward shift was reduced, whereas when the auditory stimulus ended shortly after the visual target, the visual forward shift was larger (Teramoto et al., 2010). This simple manipulation led to a strong cross-modal interaction. The visual target and the auditory stimulation were processed together, that is, the temporal information of the auditory stimulation influenced the processing of the visual target. Follow-up experiments subsequently revealed that this strong interaction only occurs if the auditory stimulation and visual target are closely temporally associated. That is, only if the onset of the auditory stimulus occurs at the same time and is presented during the entire motion of the visual target, does such a strong cross-modal interaction occur.

### Cross-modal representational momentum: Interactions between vision and touch

The question therefore arises as to whether a similar interaction would also occur in the context of visuotactile stimulation. In general, the processing of the tactile information might help to inform the temporal processing of the visual target, as observed for auditory information (Teramoto et al., 2010). Yet, for the audiovisual modality pairing, it might be reasonable from our perceptual system to relate and combine both sources of information as, in the real world, dynamic visual objects are typically accompanied by a sound (e.g., think only of a car passing by, an airplane, the “swoosh” of a fast-moving ball in sports). Such typical cross-modal correspondences of a tactile stimulus being associated with a moving visual stimulus do not occur with anything like the same regularity for the visuotactile modality combination (for an overview of research on the cross-modal correspondences, see Spence, 2011). Yet, interestingly, there are a number of examples in which tactile and auditory cues elicit similar sensory percepts, for example for the bouncing/streaming illusion (e.g., Meyerhoff et al., 2018; Meyerhoff & Scholl, 2018; Sekuler et al., 1997) or the cross-modal dynamic capture task (for a discussion, see Soto-Faraco, Spence, Lloyd, & Kingstone, 2004b). In the bouncing/streaming illusion, a simple auditory tone (presented over headphones)/short vibrotactile burst (presented on the palm) presented simultaneously to the overlap of two crossing, visual discs (presented on a computer screen), which might either be perceived as the discs streaming past or bouncing off each other, increases the frequency of the bouncing percept. Therefore, it is an open question as to whether the tactile and visual information are processed independently or, if not, exactly what kind of cross-modal interaction occurs.

**Fig. 1:**
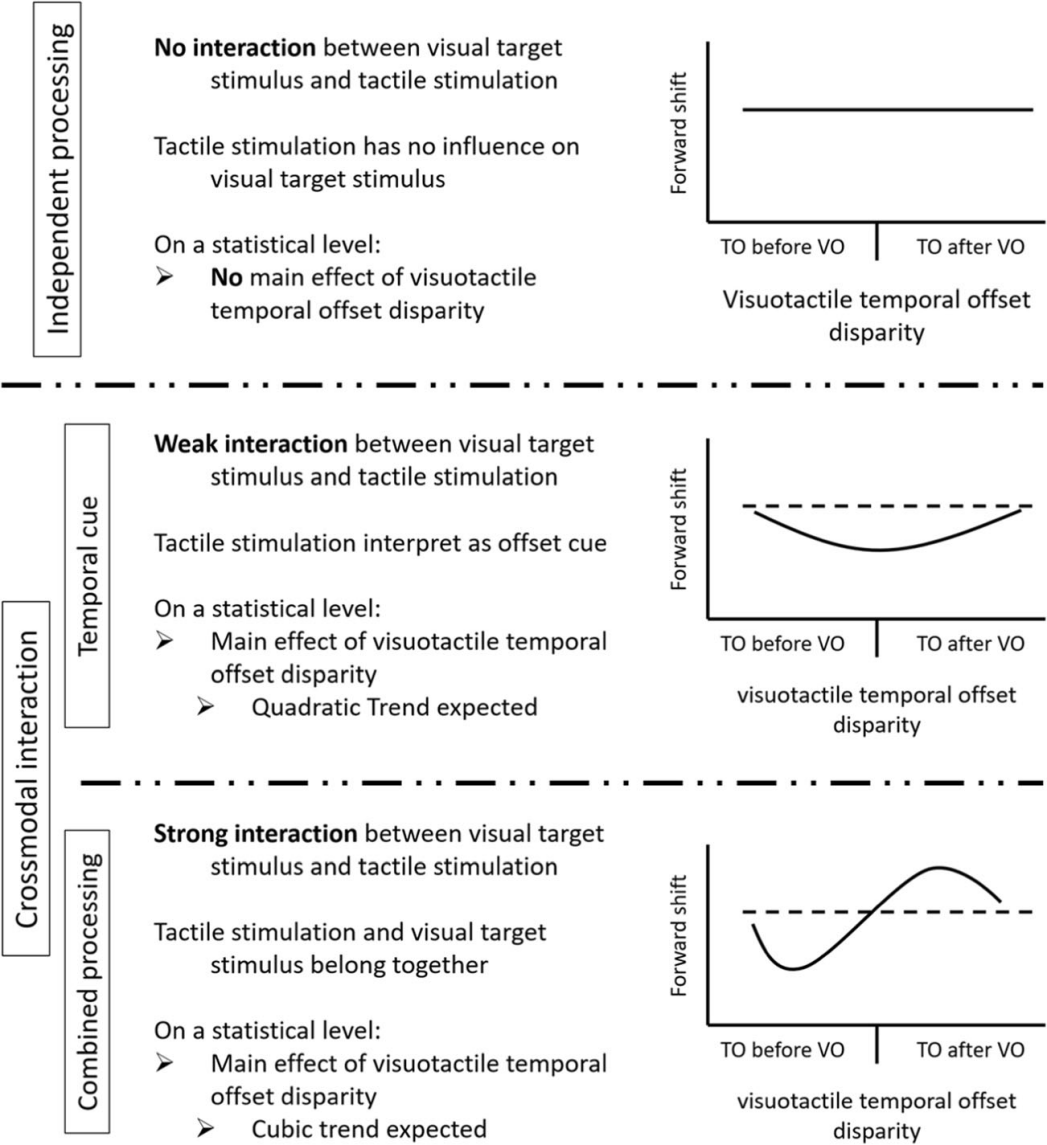
Illustration of the different theoretical hypotheses (left side) and the corresponding expected data patterns (right side). Top: Independent-processing hypothesis. Middle: Temporal-cue hypothesis. Bottom: Combined-processing hypothesis. For the expected data patterns: Solid line represents expected data pattern, dotted line represents the forward shift of the control condition (without any tactile stimulation). *TO* tactile offset, *VO* visual offset

Independent processing should manifest itself in no change of the forward shift with manipulations of the visuotactile temporal offset disparity (Fig. 1). The forward shift should not systematically vary with any changes in the disparity of the visuotactile temporal offset. In contrast, Teramoto et al.’s (2010) results with an audiovisual set-up suggest a strong interaction of the visual and auditory information, as the results directly resemble the temporal offset disparity between the visual and auditory information. Therefore, as soon as the offset disparity between the visual and tactile stimulus is uncertain in our experiment (that is, if the offset disparity is no longer obvious), the more accurate temporal perception in the tactile modality could potentially modulate the localization of the visual target. On a statistical level, such *combined processing* should manifest itself in a cubic trend in the data. Alternatively, a weak interaction of the visual and tactile data might occur (see Fig. 1 for an illustration). As the temporal processing in the tactile modality is more precise than in the visual modality (e.g., Morein-Zamir et al., 2003; Shams et al., 2000; Spence et al., 2001b; Violentyev et al., 2005), the tactile information might be taken as a temporal cue concerning the offset of the visual target (for similar ideas, see Spence & Ngo, 2012). In fact, presenting visual cues close to the offset of a visual target has been shown to decrease the magnitude of the forward shift (see Hubbard et al., 2009, who have investigated the importance of spatial target offset cues). On a statistical level, such a *temporal cue* hypothesis should manifest itself in a quadratic trend in the data, that is, with uncertainty about the offset disparity, the tactile offset might be taken as a temporal cue about the offset of the visual target and therefore reduce the forward shift by increasing participants’ temporal attention toward the offset of the visual target.

## Experiment 1

The present study investigates whether the presentation of a tactile stimulus, which is uninformative with regard to the localization of the visual target, can nevertheless still influence the perceived location of that visual target. Experiment 1 was designed to maximize the chances of a robust interaction between the visual target and the tactile stimulation, as observed previously for the audiovisual modality pairing (Teramoto et al., 2010). Therefore, the onset of the tactile stimulation and the visual target was synchronized. This condition is very similar to that of Teramoto et al.’s (2010) Experiment 1, in which the authors reported combined processing of the auditory and visual information. The temporal disparities used between the offset of the auditory stimulation and visual target in Teramoto et al.’s study ranged between -150 ms and +150 ms. Yet, prior testing by the first author (SM) indicated that the maximal temporal disparities (-150 ms and +150 ms) did not reliably give rise to a clear perception of offset disparity. Therefore, the offset disparities were increased and the participants were directly asked about the offset disparities in an additional block of trials conducted at the end of the experiment in order to assess whether a clear perception of offset disparity occurred.

### Methods

#### Participants

Visual shift scores on their own typically elicit medium to large effect sizes (dz around 0.6), therefore we aimed for at least 26 participants to find the unisensory displacement at the minimum (*α* < .05; 1-*β* > .90; power analyses were run with G-Power 3.1.9.2, option “means: difference from constant”; Faul et al., 2009). To account for possible drop-outs, N = 30 was chosen, yet, due to an organisational error, 33 participants were tested. One participant was excluded as he/she did not show any sensitivity to differences for the visuotactile temporal offset disparity (for more details, see *Results* section).^1^ The final sample (25 female, seven left-handed, mean age 21.39 years – one participant declined to enter his age) consisted of students from the University of Trier. All the participants gave written informed consent prior to participation.

#### Design

The participants were tested in a one-factorial within-participants design with the factor of *visuotactile temporal offset disparity* (-350 ms vs. -250 ms vs. -150 ms vs. -50 ms vs. ± 0 ms vs. +50 ms vs. +100 ms vs. +200 ms vs. +300 ms). A negative sign indicates that the offset of the tactile stimulus occurred before the offset of the visual target, a positive sign signifies that the offset of the tactile stimulus occurred after the offset of the visual target. Please note the fact that due to a programming error, the timing conditions before and after the offset of the visual target were not identical.^2^ Additionally, a control condition without any tactile stimulation was also assessed. In a first block of trials, the participants were asked to judge the final location of the visual target. For those localization trials, the shift scores were used as the dependent variable. Shift scores indicate the difference between the actual and the judged final location of the visual target along the horizontal x-axis (as the stimulus always moved horizontally); a negative value indicates an underestimation against the direction of motion, a positive value indicates an overestimation in the direction of motion. In a second block of trials, the participants had to judge which stimulus, the visual or the tactile, had been presented for longer. For these temporal judgment trials, the percentage of “visual stimulus longer” responses was used.

#### Apparatus and stimuli

The participants were tested in a dark, sound-attenuated laboratory. Visual stimuli were presented on a 24-in. TFT screen (1,920 × 1,200 pixel, frame rate: 60 Hz) controlled by a standard PC. The visual stimulus was a 20 × 20 pixel white square (RGB-value: 255,255,255) on a black background (RGB-value: 0,0,0). One tactor (Model C-2, Engineering Acoustic, Inc.; 3 cm in diameter, centrally located skin contactor of 0.76 cm) was attached with the help of a Velcro strap to the palm of the hand and presented the vibrotactile stimulation (~250 Hz, about 200 μm peak-to-peak amplitude). To avoid any distraction by the sound that may have been elicited by the operation of the tactor, the participants wore earplugs (noise reduction: 29 dB) and over-ear headphones over which Brown noise (simultaneously presented frequency distribution with higher intensities at lower frequencies, about 85 dB) was presented. The experiment was programmed with E-Prime 2.0, IBI SPSS statistics (Version 26) was used for data analysis of the localization scores, R (R Core Team, 2020) for the analysis of the temporal judgment scores.

#### Procedure

**Fig. 2:**
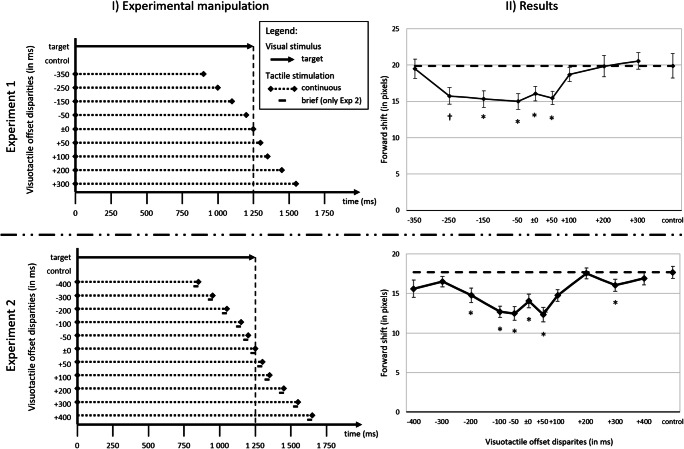
Graphical depiction of experimental manipulations as well as the results of Experiments 1 and 2. (I) Graphical depiction of the temporal relations between the onset and offset of the visual target and the continuous (Exps. 1 and 2) or brief (Exp. 2) vibrotactile stimulation. (II) Forward shift as a function of visuotactile temporal offset disparity. Error bars represent standard errors after Cousineau (2005) and the correction after Morey (2008). The dotted line indicates the forward shift in the control condition. For Experiment 2, scores are averaged across both stimulation conditions. †^/^* indicate difference from the control condition († *p* < .10; * *p* < .05)

Each trial started with a 400-ms blank screen, after which the visual target appeared on the screen and moved horizontally directly towards the center of the screen. The target was presented for 75 frames (1.25 s), with each screen refresh the target was shifted 4 pixels to the left or right, covering a distance of 300 pixels (speed of 240 pixel/s). The final location of the visual target, which had to be judged, was restricted to an 80 × 60 pixel window centered on the center of the screen. Subsequently, the starting position was 300 pixels to the left (left-to-right motion direction) or right (right-to-left motion direction) of the final location, and the y-axis value was constant throughout the whole trial, resulting in a consistent, horizontal movement of the target. Simultaneous with the onset of the visual target, the vibrotactile stimulation started (except for the no-vibration condition; for a visualization, see Fig. 2). The stimulation ended either before the visual target (-350 ms; -250 ms; -150 ms; -50 ms), simultaneous with the visual target (± 0 ms), or else after the visual target (+50 ms; +100 ms, +200 ms; +300 ms). After the offset of the visual target, a blank screen of 600 ms was presented before a mouse cursor, displayed as a crosshair, appeared at the center of the screen. The participant had to move the crosshair to the perceived final location of the visual target and indicate this location by pressing the left mouse-button. Alternatively, for the temporal judgment trials, the participants had to judge, using the left and right mouse button, whether the visual or tactile stimulus lasted longer (two-alternative forced choice task; 2AFC). After a response was given, a new trial started.

Overall, the participants worked through 288 trials, including 20 practice trials and 160 experimental trials (16 repetitions of nine visuotactile temporal offset disparity conditions and one control condition), in which the participants had to judge the final location of the visual target. After the localization judgment trials, the participants completed 108 temporal judgment trials (12 repetitions of nine visuotactile temporal offset location conditions). For the localization judgment block, the participants were given a break every 40 trials. All instructions at the beginning of the experiment, as well as between the different experimental blocks, were provided via the experimental software.

### Results

#### Temporal judgment

In a first step, the temporal judgment scores were analyzed with the frequency of “visual longer” responses as the dependent variable, in order to determine whether the offset disparity was clearly perceptible. Two of the participants indicated high “visual longer” scores when the tactile stimulus actually lasted longer and also indicated low “visual longer” scores when the visual stimulus lasted longer, strongly suggesting the usage of a reversed response mapping. Therefore, for these two participants, the scores were recoded accordingly. For each participant, a generalized linear model (GLM, accounting for the 2AFC task by using the binomial-logit function) was fitted with the continuous predictor of *visuotactile offset disparity*. Additionally, to determine whether participants were sensitive to the visuotactile temporal offset disparity, a second model was fitted to the data with only an intercept. Interestingly, for one participant, the inclusion of the visuotactile temporal offset disparity predictor had no significant effect (i.e., improvement) in the model fit (*p* = .263). This indicates that the participant was not sensitive to the changes in visuotactile temporal offset disparity, and was therefore excluded from further data analysis. All of the other participants were clearly able to perceive the change of temporal offset disparity from visuotactile offset first for the high negative values (e.g., -350, -250) to visual first for the high positive values (e.g., +200, +300).

To compare our results with existing evidence about perceived simultaneity of visual and vibrotactile stimulations (e.g.,Piéron, 1952 ; Spence et al., 2001b), the PSE, based on the GLMs with the continuous predictor of visuotactile offset disparity, was estimated for each participant. Overall, mean PSE scores corresponded to an actual offset disparity of 33 ms, with the tactile stimulus needing to be presented 33 ms longer than the visual stimulus to be perceived as having a synchronous offset. This difference was significant, *t*(31) = 2.53, *p* = .017, *d* = 0.45, and is in line with previous evidence that visual stimuli have to lead tactile stimuli in order to be perceived as simultaneous (Piéron, 1952; Spence et al., 2001b)

#### Localization judgment

The localization judgment scores were analyzed in order to determine whether and how the forward shift was influenced by the different visuotactile temporal offset disparities. A 9 (*visuotactile temporal offset disparity*: -350 ms vs. -250 ms vs. -150 ms vs. -50 ms vs. ± 0 ms vs. +50 ms vs. +100 ms vs. +200 ms vs. +300 ms) MANOVA with Pillai’s trace as a criterion and polynomial contrast coding was conducted.^3^ The contrast coding was specified in a way that the contrast weights for each factor step accounted for the spacing of the actual visuotactile temporal offset disparity steps used. The forward shift scores were used as the dependent variable. Overall, a significant forward shift (17.4 pixels) in motion direction was found, *t*(31) = 4.56, *p* < .001, *d* = 0.80, indicating the classical representational momentum effect. The main effect of visuotactile temporal offset disparity was significant, *F*(8, 24) = 2.85, *p* = .022, ɳ_p_^2^ = .487, indicating a cross-modal influence of the duration of the vibrotactile stimulus on the perceived final location of the visual target. Interestingly, of all of the eight polynomial contrasts that are possible in a nine-factorial design, the quadratic trend explains most variance and shows the strongest effect size, *F*(1, 31) = 13.50, *p* = .001, ɳ_p_^2^ = .303 (see Fig. 2). This result therefore provides strong support for the *temporal cue* hypothesis. Yet, the cubic trend contrast, although weaker than the quadratic trend, was also significant, *F*(1, 31) = 6.05, *p* = .020, ɳ_p_^2^ = .163, in line with the *combined processing* hypothesis. The linear contrast was not significant, *F*(1, 31) = 3.79, *p* = .061. Additionally, the control condition without any vibration and the condition with synchronized offset of the visual target and visuotactile stimulation (± 0 ms) were directly compared and indicated a significant difference, *t*(31) = 2.13, *p* = .041, with a weaker forward shift for the synchronized offset condition (16.1 pixels) than for the no-vibration condition (19.9 pixels). Once again, this result is in line with the *temporal cue* hypothesis.

### Discussion

The results of Experiment 1 indicate an influence of the tactile stimulation on the localization of the visual target. In contrast to evidence of no cross-modal influences of the direction of an irrelevant, tactile stimulus on the localization of a visual target (Merz et al., 2020), manipulating the temporal offset of a non-spatial tactile stimulus indicated a clear cross-modal interaction. Yet, as far as the nature of the cross-modal interaction is concerned, the results do not discriminate clearly between the *temporal cue* or the *combined processing* hypothesis. In general, the data are more in line with the *temporal cue* hypothesis than with the *combined processing* hypothesis, as indicated by the strong quadratic trend as well as the difference in the size of the forward-shift between the no-vibration condition and the synchronized vibration condition. Yet, the results also provide some evidence for the *combined processing* hypothesis, as the cubic trend was significant.

## Experiment 2

In Experiment 2, the design of Experiment 1 was repeated, but another condition was added to more explicitly contrast the two cross-modal hypotheses. That is, as in Experiment 1, we presented a condition with a constant vibrotactile stimulation, beginning with the onset of the visual target, and temporal offset disparity was once again systematically manipulated. Additionally, in a second block of trials, the vibrotactile stimulation did not start with the onset of the visual target, but was only presented as a short vibrotactile burst (about 33 ms, corresponding to two computer refresh rates). Importantly, the temporal offset of the vibrotactile stimulation (and its disparity from the offset of the visual target) was identical across the two stimulation conditions (for a visualization, see Fig. 2, I). If the *temporal cue* hypothesis is correct, the two blocks should elicit an identical pattern of data, as the temporal cue concerning the offset of the visual target is provided in both conditions. If the *combined processing* hypothesis is correct, the two blocks should differ, as only in the constant vibrotactile stimulation condition can the vibrotactile information and the visual target be associated. In contrast, in the short-burst condition, no strong interaction between the vibrotactile stimulation and the visual target should be observed (see also Teramoto et al., 2010). Therefore, no significant influence of the vibrotactile stimulation should have been observed, comparable to Teramoto and colleagues’ audiovisual results.

### Methods

#### Participants

Once again, a sample size of 30 was chosen a priori. The sample (25 female, three left-handed, mean age 21.8 years) consisted of students from the University of Trier. All the participants gave written informed consent prior to participation.

#### Design, apparatus, stimuli, and procedure

The design, apparatus, and stimuli as well as the procedure was identical to that of Experiment 1 with the following exceptions. The design was extended to include the factor *condition* (continuous vs. brief stimulation) and the factor *visuotactile temporal offset disparity* was extended to have 11 factor steps. Therefore, a 2 (*stimulation condition*: continuous vs. brief stimulation) × 11 (*visuotactile temporal offset disparity*: -400 ms vs. -300 ms vs. -200 ms vs. -100 ms vs. -50 ms vs. ± 0 ms vs. +50 ms vs. +100 ms vs. +200 ms vs. +300 ms vs. +400 ms) within-participant design was used for Experiment 2.

The factor condition was realized in two separate blocks, the order of the blocks was randomized between participants. The continuous stimulation condition was identical to the stimulation used in Experiment 1 (for a visualization, see Fig. 2). For the brief stimulation condition, the vibrotactile stimulation did not start simultaneously with the visual target, but consisted of only a short vibration burst (duration of 33 ms), corresponding to two screen refreshes. The onset of the vibrotactile stimulation was therefore dependent on the specific vibrotactile offset disparity condition (for a visualization, see Fig. 2). Importantly, the offset of the vibrotactile stimulation (and subsequently the offset disparities between the visual target and vibrotactile stimulation) was identical across both stimulation conditions. Once again, a control condition without any vibrotactile stimulation was assessed in both blocks. The temporal judgment trials were dropped for Experiment 2.

Overall, participant worked through 396 trials, this included 12 practice trials which were identical to the trials in the first experimental block. Both experimental blocks consisted of 192 trials (16 repetitions of 11 visuotactile temporal offset disparity conditions and one control condition).

### Results

A 2 (*stimulation condition*: continuous stimulation vs. brief stimulation) × 11 (*visuotactile temporal offset disparity*: -400 ms vs. -300 ms vs. -200 ms vs. -100 ms vs. -50 ms vs. ± 0 ms vs. +50 ms vs. +100 ms vs. +200 ms vs. +300 ms vs. +400 ms) MANOVA with Pillai’s trace as a criterion and polynomial contrast coding was conducted. Once again, the contrast coding was specified in a way that the contrast weights for each factor step accounted for the spacing of the actual disparity steps used. The shift scores were used as a dependent variable. Overall, a significant forward shift (14.9 pixels) in motion direction was observed, *t*(29) = 4.07, *p* < .001, *d* = 0.74, indicating the classical representational momentum effect. The main effect of visuotactile temporal offset disparity was significant, *F*(10, 20) = 6.59, *p* < .001, ɳ_p_^2^ = .767, yet, neither the main effect of stimulation condition, *F*(1, 29) < .01, *p* = .998, nor the crucial interaction between the two factors, *F*(10, 20) = 1.16, *p* = .367, was significant.^4^ This result provides clear evidence that the stimulation condition had no influence on the perceived location of the visual target. Interestingly, taking a closer look at the polynomial contrasts for the main effect of visuotactile temporal offset disparity, the quadratic trend is once again able to explain most of the variance and shows the strongest effect size, *F*(1, 29) = 18.66, *p* < .001, ɳ_p_^2^ = .392 (see Fig. 2). The cubic trend contrast, *F*(1, 29) = 1.72, *p* = .199, as well as the linear contrast, *F*(1, 29) = 2.13, *p* = .156, were not significant. Additionally, just as in Experiment 1, the control condition without any vibration and the condition with synchronized offset of the visual target and visuotactile stimulation (± 0 ms), averaged across both stimulation conditions, were directly compared. The results indicated a significant difference, *t*(29) = 3.34, *p* = .002, with a weaker forward shift for the synchronized offset condition (14.0 pixels) than in the no-vibration condition (17.7 pixels). Once again, this is in line with the *temporal cue* hypothesis.

### Discussion

In Experiment 2, the two contrasting hypotheses were more directly tested by conducting two different stimulation conditions. In one condition, the presentation of the vibrotactile stimulation began with the onset of the visual target, while in the brief stimulus condition, only a short vibrotactile burst was presented at the end. Interestingly, these two conditions did not differ, in line with the *temporal cue* hypothesis. Furthermore, the influence of the vibrotactile offset disparity was most precisely explained by the quadratic trend analysis, indicating strong support for the *temporal cue* hypothesis.

## General discussion

In two experiments, we investigated the cross-modal influence of the temporal offset (a)synchrony of tactile stimulation on the localization of a dynamic visual stimulus. When localizing the offset location of a dynamic visual stimulus, a systematic shift in motion direction, a forward shift, was observed as expected (see Hubbard, 2005, 2018, for reviews). Yet, this forward shift was significantly influenced by the temporal presentation of a spatially uninformative, tactile stimulus. More precisely, if the offset of the tactile stimulus was in close temporal proximity with the offset of the visual target (about ±200 ms), the localization of the offset of the visual target was increased, that is, the forward shift was smaller compared to the forward shift in the control condition. These results clearly indicate a cross-modal modulation of the visual target by the tactile stimulation, similar to evidence with the audiovisual modality pairing (Teramoto et al., 2010).

The two experiments also explored the nature of the visuotactile interaction. Hereby, two different hypotheses about the way in which the tactile stimulation might influence the localization of the visual target have been proposed. On the one hand, a strong cross-modal interaction might have occurred in which the information from both modalities is processed in combination to inform the final percept, as shown for the audiovisual stimulus combination (Teramoto et al., 2010). In contrast, a weak interaction between the tactile stimulation and the visual target might have occurred in which the tactile stimulation is taken as a temporal offset cue for the visual target, which subsequently led to a smaller forward shift. Similar patterns were also observed with spatial visual offset cues (Hubbard et al., 2009). The results of this study indicate a weak interaction between the tactile stimulation and the visual target. That is, the results are in line with the view that the offset of the vibrotactile stimulation has been taken as a temporal cue for the offset of the visual target.

The question arises as to how the temporal cues might have influenced the localization of the visual target. In our view, an attentional explanation is the most promising, that is, the offset of the vibrotactile stimulus might have directed attention to the possible offset of the visual target. Due to increased attention on the visual target, localization might have improved, subsequently resulting in an increase localization performance (smaller forward shifts compared to the control condition). The fact that this performance increase is more apparent when the tactile offset occurs before the visual offset (negative visuotactile temporal offset disparities; see Fig. 2) is in line with this interpretation. Yet, a strong interpretation would not predict any performance increase when tactile offset was perceived after visual offset (e.g., at offset disparities of +50 and +100 ms), but was evidenced (see *Results* section, as well as Fig. 2).^5^ This interpretation is in line with results showing that the representational momentum effect increased under dual-task conditions (when attention is not solely focused on the to-be-judged target, e.g., Hayes & Freyd, 2002; Joordens et al., 2004). Similarly, cuing the final spatial location has been shown to decrease forward shifts (e.g., Hubbard et al., 2009). Yet, the interplay between Representational Momentum and attention is far from being fully understood, even today (for discussions, see Hubbard, 2005, 2018).

Overall, the present visuotactile results stand in contrast to findings in the audiovisual modality pairing (Teramoto et al., 2010). Although for both modality combinations, a cross-modal influence of the spatially uninformative (auditory or tactile) stimulation on the localization of the dynamic visual target was found as soon as the offset-disparity was not obvious, the nature of the influence was different. That is, Teramoto and colleagues found a strong interaction between the visual target and auditory stimulation in which the auditory stimulation increased (auditory offset after visual offset) or decreased (auditory offset before visual offset) the forward shift of the visual target. This indicates a strong interaction between the auditory and visual information in their data. In line with predictions from recent accounts of multisensory integration (e.g., Ernst & Banks, 2002; Ernst & Bülthoff, 2004), the auditory modality strongly impacted the final percept as temporal (offset) processing of auditory information is typically much more precise than the temporal processing of the visual information (see also Getzmann, 2007; Shams et al., 2000). In contrast, the present visuotactile study observed, under similar conditions as used by Teramoto and colleagues, that the tactile stimulation always led to a smaller forward shift compared to the control condition, independent from which stimulus offset was first.

In our view, this indicates only a weak interaction between the visual and tactile information, and that the tactile information is only taken as a temporal offset cue about the offset of the visual target. Comparable to the audiovisual modality pairing, temporal (offset) processing in the tactile modality is faster (e.g., PSE results of Experiment 1, see also Piéron, 1952; Spence et al., 2001b) and more precise (e.g., Violentyev et al., 2005) compared to visual processing, a strong interaction should have resulted in the same data pattern as observed by Teramoto and colleagues (2010), which was not found in our study. Even more, in Experiment 2, we directly tested the temporal offset hypothesis directly by introducing an additional condition in which only a tactile offset cue was presented. Presenting a continuous vibrotactile stimulus, or else just a brief offset cue, resulted in comparable data patterns, once again supporting the *temporal cue* hypothesis, and not the *combined processing* hypothesis (for a general discussion about cross-modal grouping/organization, see Spence, 2015) .

The question arises as to why the nature of the audiovisual interaction is qualitatively different than the visuotactile interaction. In fact, cross-modal differences and processing differences for different modality pairings (such as audiovisual, visuotactile and audiotactile) have been reported, for example for cross-modal temporal adaptation and recalibration (e.g., Alais et al., 2017; see also Van der Burg et al., 2015) or the change blindness/detection paradigm (e.g., Auvray et al., 2007; Gallace et al., 2006; Gregg & Samuel, 2008; Simons & Rensink, 2005). Additionally, one possible reason for the difference between the audiovisual and visuotactile modality pairing might be the existence of many more audiovisual correspondences in the real world than visuotactile ones. For example, dynamic visual objects like a car or an airplane are typically accompanied by a sound. Yet, to the best of our knowledge, none exists for the visuotactile modality pairing. Even more closely to the experimental set-up in the Teramoto et al.’s (2010) study and our study, we are accustomed to seeing information on our computer screen which is accompanied with sounds (e.g., watching movies/video-clips). Yet, that information which is presented on our computer screen is associated with specific vibrations is not common. Perhaps these differences between the audiovisual and visuotactile modality pairing might have led to the different results. Moreover, switching attention from or to the tactile modality is more costly than switching from the auditory or visual modality (e.g., Spence et al., 2001a). Additionally, recent evidence indicates that tactile perception is different for different tactile stimulation types (e.g., electrocutaneous, vibrotactile, air puffs, touching; Hoffmann et al., 2018; Hoffmann et al., 2019), opening up the possibility that a different tactile set-up might have resulted in a different interaction between the two stimuli. Yet, if at all, and how exactly, another vibrotactile set-up might have resulted in a different data pattern is an open question to this point.

## Conclusion

In two experiments, we showed for the first time the influence of the temporal characteristics of a non-spatial, tactile stimulation on the perceived location of a dynamic visual target. Comparable to the audiovisual results that have been previously reported (Teramoto et al., 2010), the tactile information directly impacted the perceived final location of the visual target. Yet, analysis of the nature of the cross-modal interaction revealed a different pattern of results than has been reported previously for the audiovisual modality pairing. That is, the temporal characteristics of the tactile stimulation have only been taken as a temporal cue about the offset of the visual target. Yet, no strong interaction between the visual and tactile information was created, in which an elongated (shortened) presentation of the vibrotactile stimulation would have led to an increase (decrease) of the observed forward shifts, as found in audiovisual data (Teramoto et al., 2010).

## Footnotes

1 The exclusion of this participant did not significantly change the localization judgment results.

2 The programming error resulted in an asymmetry between the temporal offset disparities used in this experiment. Yet, importantly, the proportion of trials in which the tactile stimulus ended before the visual stimulus (negative offset disparities) and in which the visual stimulus ended before the tactile stimulus (positive offset disparities) were still equal. Additionally, in all of our analyses, we accounted for the different offset disparities, making an influence of this programming error on the interpretation of our results unlikely.

3 Note that all repeated-measures designs are inherently multivariate and the MANOVA has the advantage that sphericity cannot influence the results (e.g., see Tabachnick & Fidell, 2007).

4 To further support the absence of any critical effect of stimulation condition, a 2 (*stimulation condition*: continuous stimulation vs. brief stimulation) × 11 (*visuotactile temporal offset disparity*: -400 ms vs. -300 ms vs. -200 ms vs. -100 ms vs. -50 ms vs. ± 0 ms vs. +50 ms vs. +100 ms vs. +200 ms vs. +300 ms vs. +400 ms) Bayesian repeated-measures ANOVA using uniform priors was conducted in JASP (JASP Team, 2020; for the interpretation of Bayesian ANOVA, see van den Bergh et al., 2020). Here, the model with only the factor of visuotactile offset disparity was the best-fitting model, BF_M_ = 36.25. This best-fitting model was nearly 12 times more likely compared to the model with both factors (stimulation condition + visuotactile offset disparity), BF_01_ = 11.89. Even more, compared to the full model with both factors and their interaction, the model with only the factor visuotactile offset disparity was nearly 2,280 more likely, BF_01_ = 2279.12. This supports the notion that the factor stimulation condition did not have an influence on the forward-shift scores.

5 We thank an anonymous reviewer for raising this issue.
